# Nkx3.2 Promotes Primary Chondrogenic Differentiation by Upregulating *Col2a1* Transcription

**DOI:** 10.1371/journal.pone.0034703

**Published:** 2012-04-12

**Authors:** Yoshitaka Kawato, Makoto Hirao, Kosuke Ebina, Kenrin Shi, Jun Hashimoto, Yui Honjo, Hideki Yoshikawa, Akira Myoui

**Affiliations:** 1 Department of Orthopaedics, Graduate School of Medicine, Osaka University, Yamadaoka, Suita, Osaka, Japan; 2 Department of Orthopaedic Surgery, National Hospital Organization, Osaka Minami Medical Center, Kawachinagano, Osaka, Japan; 3 Medical Center for Translational Research, Osaka University Hospital, Yamadaoka, Suita, Osaka, Japan; University of Bari & Consorzio Mario Negri Sud, Italy

## Abstract

**Background:**

The Nkx3.2 transcription factor promotes chondrogenesis by forming a positive regulatory loop with a crucial chondrogenic transcription factor, Sox9. Previous studies have indicated that factors other than Sox9 may promote chondrogenesis directly, but these factors have not been identified. Here, we test the hypothesis that Nkx3.2 promotes chondrogenesis directly by Sox9-independent mechanisms and indirectly by previously characterized Sox9-dependent mechanisms.

**Methodology/Principal Findings:**

C3H10T1/2 pluripotent mesenchymal cells were cultured with bone morphogenetic protein 2 (BMP2) to induce endochondral ossification. Overexpression of wild-type Nkx3.2 (WT-Nkx3.2) upregulated glycosaminoglycan (GAG) production and expression of type II collagen α1 (*Col2a1*) mRNA, and these effects were evident before WT-Nkx3.2-mediated upregulation of *Sox9*. RNAi-mediated inhibition of *Nkx3.2* abolished GAG production and expression of *Col2a1* mRNA. Dual luciferase reporter assays revealed that WT-Nkx3.2 upregulated *Col2a1* enhancer activity in a dose-dependent manner in C3H10T1/2 cells and also in N1511 chondrocytes. In addition, WT-Nkx3.2 partially restored downregulation of GAG production, Col2 protein expression, and *Col2a1* mRNA expression induced by *Sox9* RNAi. ChIP assays revealed that Nkx3.2 bound to the *Col2a1* enhancer element.

**Conclusions/Significance:**

Nkx3.2 promoted primary chondrogenesis by two mechanisms: Direct and Sox9-independent upregulation of *Col2a1* transcription and upregulation of *Sox9* mRNA expression under positive feedback system.

## Introduction

During endochondral ossification, chondrogenesis is executed in multiple steps [1]. In the early steps of chondrogenesis, the Sox9 transcription factor is a crucial inducer of the expression of chondrocyte-specific genes [2], [3]. However, the latter steps of chondrogenesis are regulated by Runx-family transcription factors [4], [5]. We recently reported that hypoxia (5% oxygen tension) promotes chondrogenesis and glycosaminoglycan (GAG) production and that it suppresses hypertrophy of chondrocytes and osteoblastic differentiation in C3H10T1/2 cell culture [6]. Nkx3.2 (also known as Bapx1), a member of the NK homeobox gene family, is a transcriptional repressor that regulates Runt related transcription factor 2 (Runx2) expression as part of a tight positive regulatory system that includes Sox9; this system is initiated by Sonic hedgehog (Shh) [7], [8], [9], [10]. Lassar's group demonstrated that Nkx3.2 also promotes primary chondrogenic differentiation using primary presomitic mesodermal explants [7], but a direct, Sox9-independent relationship between Nkx3.2 and type II collagen α1 (Col2a1), a marker of primary chondrogenic differentiation, has not been reported.

In humans, a rare form of skeletal dysplasia, called spondylo-megaepiphysial-metaphysial dysplasia (SMMD), is caused by homozygous inactivating mutations in the *NKX3.2* gene [11]. The skeletal phenotypes seen in SMMD patients are morphologically similar to those observed in *Nkx3.2* null mice [12]. The human and mouse *Nkx3.2* mutations are associated not only with severe dysplasia of vertebral column, but also arrested development of intervertebral discs, which is similar to that seen in *Col2a1* null mice [13]. Therefore, we hypothesized that there must be a Sox9-independent mechanism for Nkx3.2 to promote primary chondorogenesis by activating *Col2a1* transcription.

Here, we used the C3H10T1/2 pluripotent mesenchymal cell line and the N1511 murine chondrocyte cell line and demonstrated that Nkx3.2 promotes primary chondrogenesis during endochondral ossification by directly activating *Col2a1* transcription.

## Materials and Methods

### Cell Culture and Analysis for Chondrogenic Differentiation

C3HT101/2 cells were purchased from RIKEN Bio-Resource Center (Saitama, Japan) and were cultured in Dulbecco's modified Eagle's medium (Invitrogen, San Diego, CA). The N1511 murine chondrocyte cell line [14], which was kindly donated by Dr. Hideto Watanabe and Dr. Nobuhiro Kamiya, was also cultured in Dulbecco's modified Eagle's medium. When cultures were approximately 90% confluent, recombinant human bone morphogenic protein 2 (rh-BMP2), which was donated by Osteopharma Inc. (Osaka, Japan), was added to stimulate chondrogenesis. To evaluate chondrogenic differentiation, C3H10T1/2 cells were fixed with 10% formalin and then washed with 0.1N HCl in distilled water; washed cells were stained for 60 min with alcian blue solution, alcian blue 8GX (Sigma, St. Louis, MO) and rinsed twice with 0.1 N HCl to remove any unbound dye. To quantify GAG synthesis, alcian blue is extracted by 4 M guanidine-HCl overnight at 4°C. Absorbance values are read at 600 nm after temperature equilibration.

### Proliferation assay

C3H10T1/2 cells were cultured in 96-well plates at a concentration of 1.0×10^4^ cells/cm^2^, and they were stimulated with rh-BMP2 (300 ng/ml). Cell proliferation was assessed using the Cell Counting Kit-8 assay system (Dojindo, Kumamoto, Japan), according to the manufacturer's instructions.

### Western blot analysis

Western blot analyses were performed using whole cell lysates. For the blots, 20 µg of each sample was applied. The blots were first incubated with anti-mouse collagen II antibodies (R&D Systems Inc., Minneapolis, MN) and then with horseradish peroxidase-conjugated anti- sheep IgG antibodies (R&D Systems Inc., Minneapolis, MN). Anti-mouse *β*-actin rabbit antibodies and horseradish peroxidase-conjugated anti-rabbit IgG antibodies were purchased from Cell Signaling Technology (Tokyo, Japan).

### RNA interference and overexpression of *Nkx3.2* and *Sox9*

Commercially synthesized small interfering RNA (siRNA) and manufacturer's protocols were used for the RNAi experiments (B-Bridge International, Inc., Cupertino, CA). The sequences of sense strands of siRNA targeting *Nkx3.2* and *Sox9* mRNA and that of the negative control siRNA are shown in Table 1. The wild-type Sox9 (WT-Sox9) construct was donated by the Department of Biochemistry, Osaka University Graduate School, Faculty of Dentistry. The wild-type Nkx3.2 (WT-Nkx3.2) plasmid was constructed by inserting a full-length *Nkx3.2* cDNA into the pBApo-CMV Neo vector purchased from Takara Bio Inc., Otsu, Japan. When cultures were approximately 70% confluent, 10 nM of Nkx3.2 or Sox9 siRNA were transfected with 10 µM Lipofectamine RNAiMAX (Invitrogen). After 24-h transfection, MOCK plasmid or WT-Nkx3.2/Sox9 was transiently transfected by using FuGENE®6 transfection reagent (Roche, Indianapolis, IN) according to the manufacture's recommendation. After another 24-h culture (day 0), cells were continuously stimulated with BMP-2 (300 ng/ml) and cultured with the medium change at 3 days intervals.

**Table 1:** Sequences of sense strands of siRNA targeting *Nkx3.2*, *Sox9* mRNA and of siRNA control.

Target gene	Oligonucleotide	Sequence (5′→3′ )
Negative control	Sense	ATCCGCGCGATAGTACGTA
*Nkx3.2*	Sense	CCAAGGACCUGGAGGAGGATT
*Sox9*	Sense	GCCAGGUGCUGAAGGGCUATT

### Reporter Constructs and Luciferase Reporter Assay

Four tandem copies of a 48-bp chondrocyte-specific enhancer segment from the *type II collagen α1* (*Col2a1*) gene were synthesized as previously reported [15] and inserted into the pGL3-Promoter vector (Promega, Madison, WI); this construct was designated 4Col2E-Luc. A GTGAAT motif was deleted from this 48-bp enhancer segment to generate a 42-bp segment; an array of four tandem copies of this 42-bp segment was synthesized and inserted into the pGL3-Promoter vector purchased from UNITECH (Chiba, Japan). This construct was designated D4Col2E-Luc. In reporter assays, cells were transiently transfected with 0.4 µg of the 4Col2E-Luc or the D4Col2E-Luc construct and 0.01 µg of the TK-Renilla luciferase construct (TK *Renilla*) (Promega). Luciferase activity was measured using a Dual Luciferase assay kit (Promega) and luminometer (Berthold Technologies, Bad Wildbad, Germany), and reporter construct activity was normalized by comparison with activity from the *Renilla* luciferase construct. All experiments were performed in triplicate.

### Chromatin immunoprecipitation (ChIP)

Chromatin immunoprecipitation (ChIP) was performed using an EZ ChIP kit (Millipore, Billerica, MA). Anti-Nkx3.2 mouse antibodies, anti-Sox9 mouse antibodies, and normal rabbit immunoglobulin G (IgG) were all purchased from Santa Cruz Biotechnology, Inc. (Santa Cruz, CA). Qualitative PCR conditions were as follows: 1 cycle at 95°C for 2 min; 35 cycles at 95°C for 30 s, 60°C for 30 min, and 72°C for 30 min; and 1 cycle at 72°C for 7 min. Products were electrophoresed on a 3% agarose gel. ChIP primers targeting the *Col2a1* enhancer are shown in Table 2.

**Table 2:** Primer sequences used in ChIP analysis of the *Col2a1* enhancer.

Primer	Sequence (5′→3′ )
Forward	GGGAGACCTCAGTCCTCCTT
Reverse	AGAAAGGAGCCAACGCTGTA

### Standard and Quantitative RT-PCR

First-strand cDNA was synthesized using SuperScript III RNase H® reverse transcriptase (Invitrogen). Standard RT-PCR was performed using Ex Taq (Takara Bio Inc.). Quantitative RT-PCR was performed using the Roche Applied Science Light Cycler system. The SYBR Green assay, with which each cDNA sample was evaluated in triplicate 20-µl reactions, was used for all target transcripts. Expression values were normalized to *GAPDH* expression. The sequence of the primers used for standard and quantitative RT-PCR assays, are shown in Table 3.

**Table 3:** Primer sequences used in PCR assays.

gene	Primer	Sequence (5′→3′)
*Nkx3.2*	Forward	AACCATCAGCGCTACCTGTC
	Reverse	CTTTACGGCCACTTTCTTGG
*Col2a1*	Forward	CCTGTCTGCTTCTTGTAAAAC
	Reverse	AAAAAATACAGAGGTGTTTGACACAGA
*Sox9*	Forward	ATGAATCTCCTGGACCCCTT
	Reverse	AACTTTGCCAGCTTGCACGT
*Runx2*	Forward	GCTTGATGACTCTAAACCTA
	Reverse	AAAAAGGGCCCAGTTCTGAA
*GAPDH*	Forward	TGAACGGGAAGCTCACTGG
	Reverse	TCCACCACCCTGTTGCTGTA

### Statistical analysis

All data are expressed as the mean ± standard deviation (SD) of a minimum of three replicate measurements. Differences between groups were assessed using the ANOVA test. Any P value<0.05 was considered statistically significant.

## Results

### Overexpression of Nkx3.2 promotes and inhibition of *Nkx3.2* suppresses chondrogenic differentiation and GAG production in C3H10T1/2 cell culture

**Figure 1:**
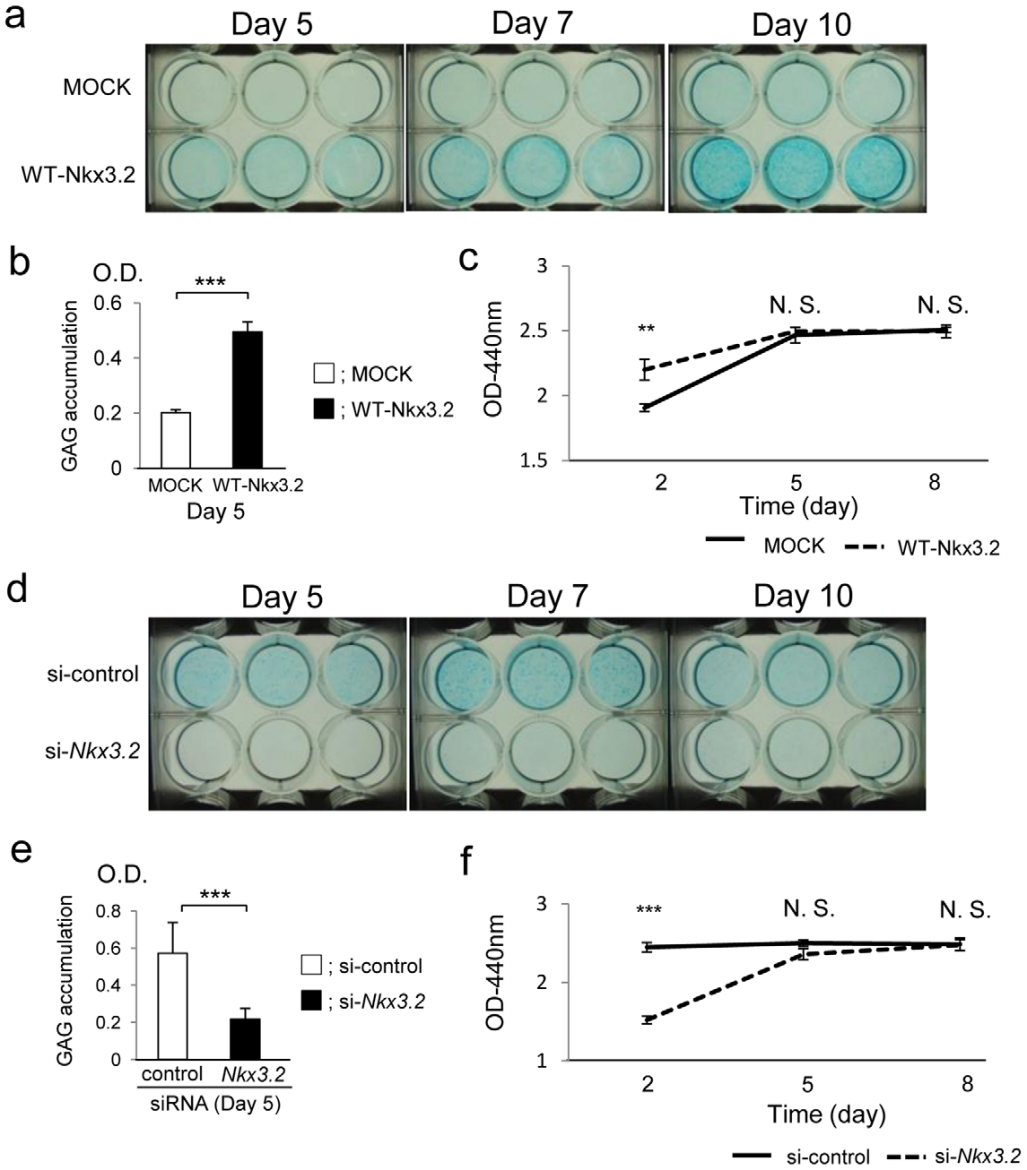
Overexpression of Nkx3.2 and inhibition of *Nkx3.2* affected GAG production and cell proliferation in C3H10T1/2 cell culture. **a** After 24-h transfection with MOCK plasmid (upper wells) or WT-Nkx3.2 (lower wells) (day 0), C3H10T1/2 cells were stimulated with BMP-2 (300 ng/ml) for 5, 7, or 10 days. At each time point, cells were stained with alcian blue. **b** Quantification of GAG synthesis by C3H10T1/2 cells on day 5. Data are shown as mean ± S.D. (n = 3). ***P<0.001. **c** Proliferation of C3H10T1/2 cells transfected with MOCK or WT-Nkx3.2 plasmid. Transfected C3H10T1/2 cells were stimulated with BMP-2 (300 ng/ml) for 8 days. Cell proliferation was assayed on day 2, 5, and 8. **P<0.01; *N.S.*, not significant. **d** After a 48-h transfection with negative control siRNA (upper wells) or with *Nkx3.2* siRNA (lower wells) (day 0), C3H10T1/2 cells were stimulated with BMP-2 (300 ng/ml) for 5, 7, or 10 days. At each time point, cells were stained with alcian blue. **e** Quantification of GAG synthesis by C3H10T1/2 cells on day 5. Data are means ± S.D. (n = 3). ***P<0.001. **f** Proliferation of C3H10T1/2 cells transfected with si-control or si-*Nkx3.2*. Transfected C3H10T1/2 cells were stimulated with BMP-2 (300 ng/ml) for 8 days. Cell proliferation was assayed on days 2, 5, and 8. ***P<0.001; *N.S.*, not significant.

**Figure 2:**
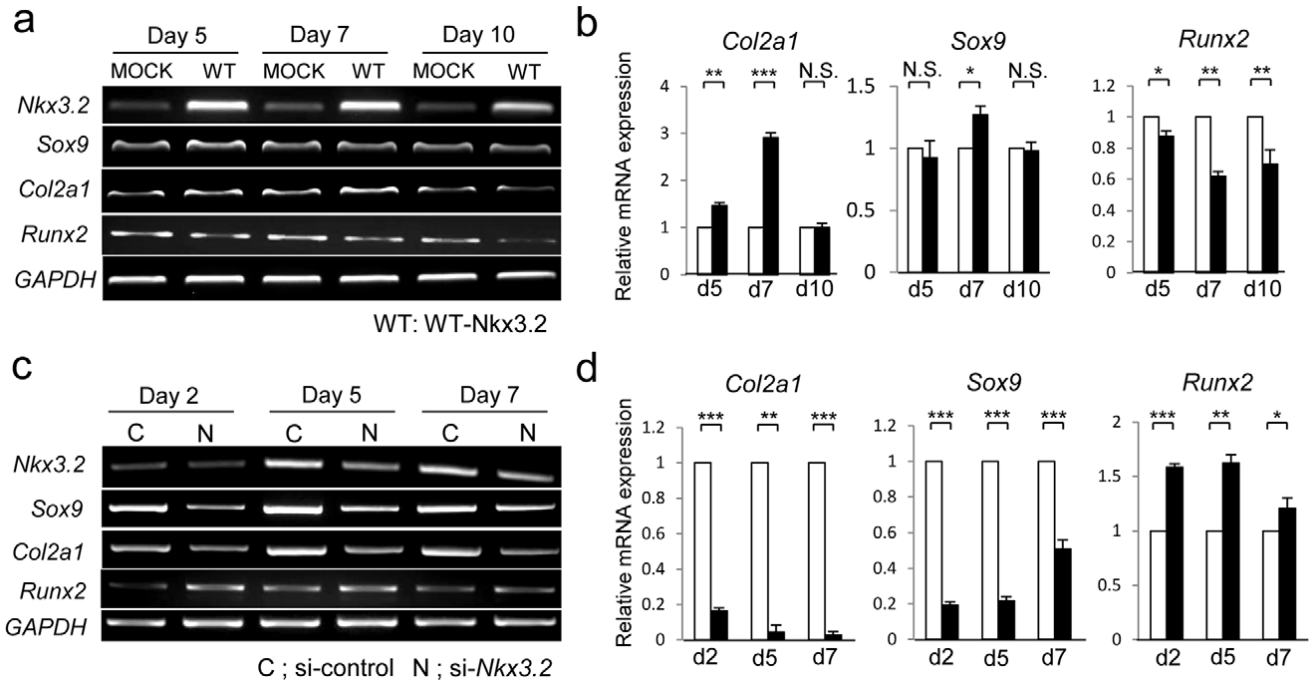
Overexpression of Nkx3.2 and inhibition of *Nkx3.2* affected mRNA expression of chondrogenic differentiation markers in C3H10T1/2 cell culture. **a** After 24-h transfection with MOCK plasmid or WT-Nkx3.2, C3H10T1/2 cells were stimulated with BMP-2 (300 ng/ml) for 5, 7, and 10 days and total RNA was extracted at each time point. RT-PCR was used to assess *Nkx3.2*, *Sox9*, *Col2a1*, *Runx2*, and *GAPDH* mRNA expression. **b** Real-time PCR for *Col2a1*, *Sox9*, *Runx2*, and *GAPDH* mRNAs were also performed. Values are normalized to the level of *GAPDH* mRNA. Data are presented as means ± S.D. (n = 3). *P<0.05; **P<0.01; ***P<0.001; *N.S.*, not significant. **c** After a 48-h transfection with negative control siRNA or with *Nkx3.2* siRNA (day 0), C3H10T1/2 cells were stimulated with BMP-2 (300 ng/ml) for 2, 5, and 7days. At each time point, total RNA was extracted. RT-PCR was used to assess *Nkx3.2*, *Sox9*, *Col2a1*, *Runx2*, and *GAPDH* mRNA expression. **d** Real-time PCR was also used to assess *Col2a1*, *Sox9*, *Runx2*, and *GAPDH* mRNA expression. Values are normalized to the level of *GAPDH* mRNA. Data are presented as means ± S.D. (n = 3). *P<0.05; **P<0.01; ***P<0.001.

On days 5, 7, and 10, GAG production was higher in C3H10T1/2 cells overexpressing Nkx3.2 than in control cells (Fig. 1a, b). Expression of *Col2a1* mRNA was upregulated on days 5 and 7, and *Runx2* mRNA was downregulated on days 5, 7, and 10 in cells overexpressing Nkx3.2. However, *Sox9* expression was not upregulated until day 7 (Fig. 2a, b). Overexpression of Nkx3.2 upregulated cell proliferation in C3H10T1/2 cells on day 2, but no differences in cell proliferation were observed between control cells and overexpressing cells after day 5 (Fig. 1c). Inhibition of *Nkx3.2* using RNAi clearly suppressed GAG production on days 5, 7 and 10 (Fig. 1d, e) and expression of *Col2a1, and Sox9* mRNA, while upregulated *Runx2* expression on days 2, 5, and 7 (Fig. 2c, d). Inhibition of *Nkx3.2* downregulated the proliferation of C3H10T1/2 cells on day 2, but no differences in cell proliferation were observed after day 5 (Fig. 1f).

### Nkx3.2 binds to the 48-bp chondrocyte-specific enhancer segments of *Col2a1* and upregulates *Col2a1* transcription

**Figure 3:**
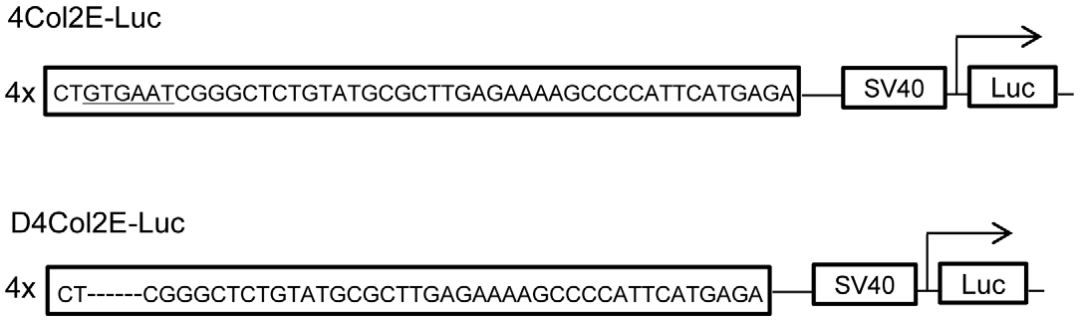
Schematic of reporter constructs. 4Col2E-Luc has four tandem copies of a 48-bp chondrocyte-specific enhancer segment from the *type II collagen α1* (*Col2a1*) gene. D4Col2E-Luc construct has four tandem copies of a 42-bp version of the 48-bp segment; a GTGAAT motif was deleted from the 48-bp chondrocyte-specific enhancer segment from *Col2a1* to generate the 42-bp version.

**Figure 4:**
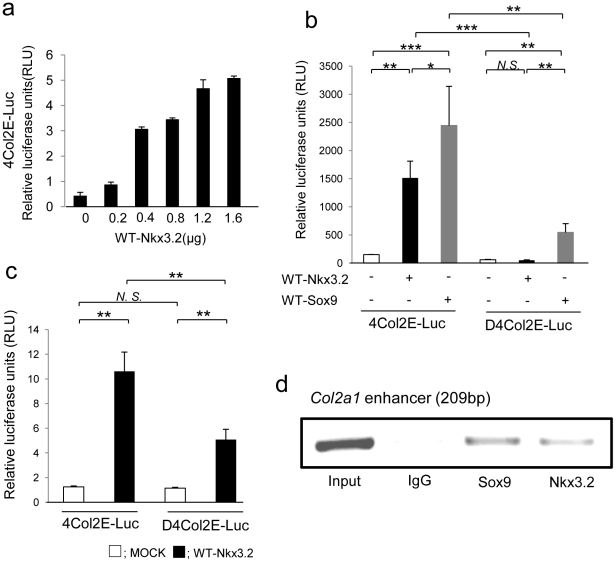
Nkx3.2 upregulated 48-bp *Col2a1* enhancer activity in C3H10T1/2 cells (**a** and **b**) and in N1511 cells (**c**) and binds to 48-bp *Col2a1* enhancer (**d**). **a** Transient transfection of Nkx3.2 showed does-dependent activation of 4Col2E-Luc. **b** Transcriptional activity of 4Col2E-Luc and D4Col2E-Luc using WT-Nkx3.2. Activation by WT-Sox9 is shown as a positive control. *P<0.05; **P<0.01; ***P<0.001; *N.S.*, not significant. **c** Transient transfection of Nkx3.2 resulted in activation of the 4Col2E-Luc construct in N1511 cells. In contrast, the D4Col2E-Luc construct was not activated by Nkx3.2 overexpression. **P<0.01; *N.S.*, not significant. **d** Chromatin immunoprecipitation was performed using Nkx3.2, Sox9, and control antibodies in C3H10T1/2 cells. Products were obtained from PCR of immunoprecipitated chromatin with primers for the *Col2a1* enhancer. The sizes of the products are indicated. The first lane in each panel shows the PCR product obtained with chromatin input. Data are representative of three independent experiments.

To assess the effects of Nkx3.2 on *Col2a1* enhancer activity, we performed a dual luciferase activity assay. The D4Col2E-Luc construct carried a deleted version of the enhancer carried in the 4Col2E-Luc construct; specifically, the enhance repeats in D4Col2E-Luc lack a GTGAAT motif (Fig. 3). WT-Nkx3.2 upregulated *Col2a1* enhancer activity in a dose-dependent manner (Fig. 4a). Next, we compared the activity from the 4Col2E-Luc and D4Col2E-Luc constructs in the presence of WT-Nkx3.2 or WT-Sox9 (Fig. 4b, c). Both WT-Nkx3.2 and WT-Sox9 clearly upregulated the transcriptional activity of 4Col2E-Luc (Fig. 4b). Moreover, WT-Nkx3.2 upregulated transcriptional activity of 4Col2E-Luc not only in C3H10T1/2 cells but also in N1511 chondrocytes (Fig. 4c). In contrast, WT-Nkx3.2 did not upregulate activity of the D4Col2E-Luc construct (Fig. 4b, c). We also used the ChIP assay to determine whether Nkx3.2 bound to the 48 bp *Col2a1* enhancer element, and found that Nkx3.2 did bind to this enhancer element (Fig. 4d).

### Overexpression of Nkx3.2 partially restored downregulation of GAG production, Col2 protein expression, *and Col2a1* mRNA expression induced by si-*Sox9*

**Figure 5:**
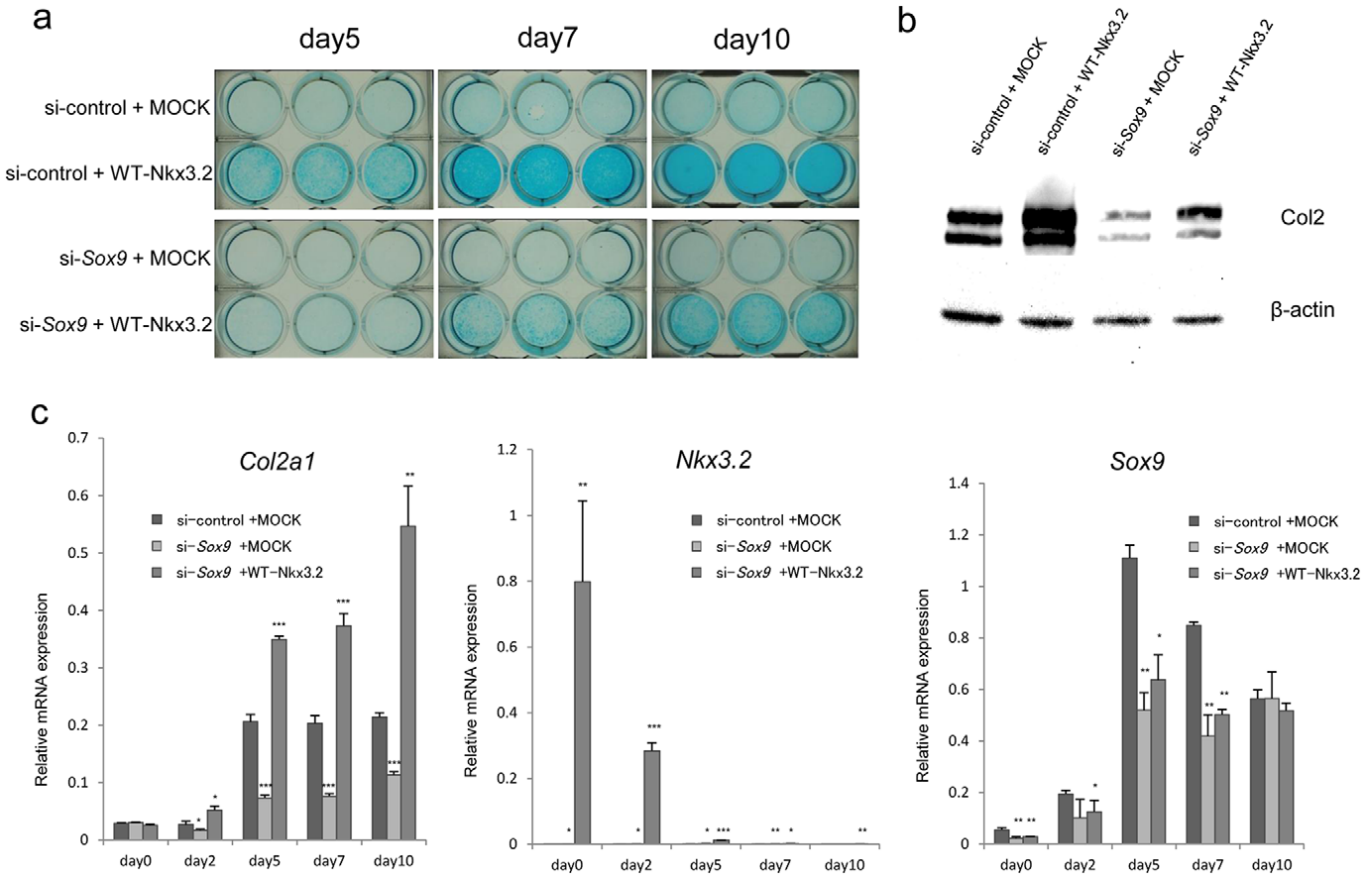
Overexpression of Nkx3.2 partially restored downregulation of GAG production (**a**), Col2 protein expression (**b**), *and Col2a1* mRNA expression (**c**) induced by si-*Sox9* in C3H10T1/2 cell culture. **a** After 24-h transfection with si-control (upper panels) or si-*Sox9* (lower panels), MOCK plasmid (upper wells) or WT-Nkx3.2 (lower wells) were transfected. After another 24-h culture (day 0), cells were stimulated with BMP-2 (300 ng/ml) for 5, 7, and 10 days. At each time point, cells were stained with alcian blue. **b** Western blot of Col2 on day 7. Overexpression of Nkx3.2 partially restored Col2 protein expression downregulated by si-*Sox9*. **c** Total RNA was extracted at each time point. Real-time PCR was used to assess *Col2a1*, *Nkx3.2*, *Sox9*, and *GAPDH* mRNA expression. Values are normalized to the level of *GAPDH* mRNA. Data are presented as means ± S.D. (n = 3). P values are evaluated v.s. si-control+MOCK group. *P<0.05; **P<0.01; ***P<0.001; no marks represents no significant differences.

To assess Sox9-independent upregulation of *Col2a1* trascription by Nkx3.2, we performed *Sox9* inhibiting experiments using RNAi. On days 5, 7, and 10, GAG production was downregulated by si-*Sox9* compared to si-control (Fig. 5a). Si-*Sox9* downregulated *Col2a1* mRNA expression from day 2 and *Sox9* mRNA expression from day 0, while *Sox9* mRNA was restored on day 10 (Fig. 5c). Overexpression of Nkx3.2 upregulated its mRNA expression from day 0 to day 10, and partially restored downregulation of GAG production and Col2 protein expression induced by si-*Sox9* (Fig. 5a, b). Overexpression of Nkx3.2 with si-*Sox9* upregulated *Col2a1* mRNA expression even more than that of si-control+MOCK, and also tended to restore *Sox9* mRNA expression downregulated by si-*Sox9* suggesting positive feedback of Nkx3.2 to Sox9 (Fig. 5c).

## Discussion

Previously, we presented the hypothesis that factors other than Sox9 directly promote chondrogenic differentiation [6]. Here, we tested our hypothesis by assessing whether Nkx3.2, a well-known transcriptional repressor of *Runx2*, directly regulates *Col2a1*, a marker of chondrogenesis [8], [16].

Downregulation of *Nkx3.2* in mouse embryos results in severe skeletal dysplasia and death [10]. Interestingly, arrested development of the intervertebral discs in *Nkx3.2* null mice [12] is clearly reminiscent of the defects observed in *Col2a1* null mice [12]. These findings led us to suppose that *Nkx3.2* may play a crucial role in the regulation of *Col2a1* expression.

Previously, it has been reported that *Nkx3.2* gene expression begins somewhat earlier than *Sox9* expression during chondrogenic differentiation in C3H10T1/2 cells [8]. Here, we found that exogenous Nkx3.2-mediated upregulation of a chondrogenic gene, *Col2a1*, occurred earlier than Nkx3.2-mediated upregulation of *Sox9* (Fig. 2a, b). In addition, overexpression of Nkx3.2 partially restored downregulation of GAG production, Col2 protein expression, *and Col2a1* mRNA expression induced by si-*Sox9*, suggesting Sox9-independent mechanism for upregulation of *Col2a1* trascription by Nkx3.2.

Nkx3.2 reportedly binds to a HRAGTG motif [17]. Notably, we discovered a GTGAAT motif in the *Col2a1* enhancer element, and GTGAAT is the reverse of HRAGTG. Therefore, we speculated that Nkx3.2 may bind this reverse motif and regulate *Col2a1* transcription. We assessed the effects of overexpression of Nkx3.2 or Sox9 on *Col2a1* enhancer activity using the 4Col2E-Luc and D4Col2E-Luc constructs. These assays clearly demonstrated that Nkx3.2 overexpression upregulated 4Col2E-Luc activity, but Nkx3.2 overexpression had less of an effect than Sox9 overexpression. In addition, D4Col2E-Luc transcriptional activity was not upregulated by WT-Nkx3.2 overexpression, but WT-Sox9 overexpression resulted in upregulation of D4Col2E-Luc. We suppose that WT-Sox9 was able to upregulate D4Col2E-Luc because the 42-bp repeats in D4Col2E-Luc each contained three Sox-9 binding sites [15], [18]. Taken together, these results indicate that the GTGAAT motif in the 48-bp *Col2a1* enhancer was important for the transcriptional activation mediated by either Nkx3.2 or Sox9. Finally, results of ChIP assays confirmed that Nkx3.2 did bind to the 48-bp *Col2a1* enhancer.

Reportedly, a trio of Sox proteins (Sox9, L-Sox5, and Sox6) is crucial to upregulation of *Col2a1* transcription [18]. However, Sox9-dependent transcriptional regulation in chondrogenesis requires cofactors in addition to these Sox proteins, such as p300/CREB-binding protein (CBP), peroxisome proliferator-activated receptor γ (PPAR-γ) coactivator-1α (PGC-1α), and Smad3 [19], [20], [21], [22], [23]. Therefore, we first assumed that Nkx3.2 and some Sox family proteins cooperate to upregulate *Col2a1* enhancer activity. Meanwhile, our preliminary experiment showed that overexpression of Nkx3.2 and Sox9 together was more effective than overexpression of Nkx3.2 alone, but less effective than overexpression of Sox9 alone, in upregulating *Col2a1* enhancer activity (data not shown). Finally, exogenous Nkx3.2 partially restored GAG production, Col2 protein expression, *and Col2a1* mRNA expression downregulated by si-*Sox9*. Meanwhile, exogenous Nkx3.2 also tended to restore Sox9 after day 2, and this positive feedback may partially contribute to the restoration of GAG production and *Col2a1* mRNA expression by Sox9-dependent pathway. To obtain stronger downregulation of Sox9, we performed higher dose of si-*Sox9* experiments, which resulted in decreased cell viability of C3H10T1/2 cells. Based on these findings, it seemed that Nkx3.2 could induce chondrogenesis by Sox9-independent mechanisms in C3H10T1/2 cells, but that it was less effective than Sox9.

Nkx3.2 expression is maintained in proliferative chondrocytes, and Nkx3.2 promotes viability of these cells by constitutively activating RelA during endochondral ossification [24]. Therefore, we first assumed that Nkx3.2 also controls the viability of C3H10T1/2 cells stimulated with BMP2. As shown in Fig. 1c and Fig. 1f, overexpression of Nkx3.2 upregulated and inhibition of *Nkx3.2* downregulated C3H10T1/2 cell proliferation on day 2, but there was no significant difference from days 5 to 8. Our quantitative RT-PCR data showed that expression of *type X collagen α1 (Col10a1)*, a hypertrophic chondrocyte marker, was detected from day 5 (data not shown). Although it is difficult to demonstrate that the phenotype of C3H10T1/2 cells stimulated with BMP2 for 2 days is similar to that of proliferative chondrocyte, it is plausible that Nkx3.2 affected cell proliferation in proliferative chondrocyte, while its effect seemed to vanish in hypertrophic chondrocytes.

This study has several limitations. We used only two cell lines in this study; therefore, we must investigate the interactions between Nkx3.2 and Col2a1 transcription in primary cell culture in future studies. In addition, we attempted to detect direct binding of Nkx3.2 to the 48-bp *Col2a1* enhancer using electrophoresis mobility shift assay and nuclear extracts of C3H10T1/2 cells transfected with WT-Nkx3.2 and 48-bp *Col2a1* probes and antibody targeting Nkx3.2. However, we could not detect a clear supershift; this result may have been due to some unsolvable difficulties (data not shown). Further confirmation is required to determine whether Nkx3.2 binds directly to the 48-bp *Col2a1* enhancer.

In conclusion, we have demonstrated for the first time that the Nkx3.2 transcription factor promoted primary chondrogenesis via binding to *Col2a1* enhancer element followed by upregulation of *Col2a1* transcription by both Sox9-dependent and Sox9-independent mechanisms.
